# Optimizing the technological and informational relationship of the health care process and of the communication between physician and patient. The impact of Preventive Medicine and social marketing applied in Health Care on youth awareness–Preliminary study

**Published:** 2011-02-25

**Authors:** CM Petrescu, IR Gheorghe, GD Petrescu

**Affiliations:** * ‘Carol Davila’ University of Medicine and Pharmacy, BucharestRomania; ** Academy of Economic Studies, BucharestRomania

**Keywords:** salty mealsy, fat meals, workout exercises, caffeine consumption, tobacco consumption

## Abstract

**Rationale.** In this case study we wanted to find out the impact of Preventive Medicine and implicitly social marketing upon young students with the average age of 19, belonging to the academic environment in Romania.

**Methods** The study lasted one month and consisted of a questionnaire that was conceived and applied to 304 adolescents. The questionnaire contained demographic and personal information, such as age, origin, gender, marital status and some questions related to the respondents' attitude towards several issues that are inserted in the preventive medicine discipline, such as the date of their last consultation, if the respondents were registered to a family physician, suffered from chronic diseases, what was the rate of doing physical exercises, if they ate salty and fat meals, if they were on a diet, their rate of alcohol, caffeine and tobacco consumption.

**Discussion.** Ostium secundum defect is the most common type of atrial septal defect and accounts for 60–70% of all cases. The malformation often goes unnoticed for decades because symptoms may be absent and because physical signs are subtle. Symptoms usually take 30–40 years to develop. They are the consequences of pulmonary hypertension, atrial tachyarrhythmias and, sometimes, associated mitral valve disease.

**Results**The panel was made up of more female respondents than male, with the average age rate of 19, who had medical consultations in the last 3 months, are included in the evidence of a family physician, had no chronic diseases, usually do workout exercises moderately, are not on a diet and have 3 meals per day. The meals are medium salty and rarely rich in fats. They drink 2 cups of tea per day and are non–alcohol drinkers and non-smokers.

**Discussion**After applying several statistical tests to find a correlation between our variables, we reached the conclusion that even if the results are encouraging; there is no correlation between the impact of Preventive Medicine and the respondents' health behavior.

**Abbreviations:** PM–Preventive Medicine, UNICEF–United Nations Children's Fund

## Introduction

This research consists of three major studies: the preliminary study that deals with Preventive Medicine, the primary study that analyses the diagnosis seen from the point of view of the patient and the final study that encompasses the physicians' opinions when making a diagnosis.

We wanted to find out if there is a connection between the health behavior of our respondents and Preventive Medicine among young people in Romania.

Preventive medicine (PM) is a medical specialty concerned with the health of populations. The aim of PM is to preserve good health, to prevent disease, injury and disability and to facilitate early diagnosis and treatment of illness.[1]

PM is composed of the following specialties:[2]

- Biostatistics which includes the application of the biostatistical principles and methodology
- Epidemiology which is based on population medicine and research
- Health Services management and administration that might include: developing, assessing and assuring health policies; planning, implementing, directing, budgeting and evaluating the population' health and disease management programs; and also using legislative and regulatory processes to enhance health
- Control of environmental factors that may adversely affect health
- Control and prevention of occupational factors that may adversely affect health safety
- Clinical preventive medicine activities, including measures to promote health and prevent the occurrence, progression and disabling effects of disease and injury
- Assessment of social, cultural and behavioral influence on health.

PM also covers patient interviews and testing to detect risk factors; sanitary measures in homes, communities, and medical facilities; patient education; and diet and exercise programs as well as preventive drugs and surgery. Physicians devoted to health promotion and disease prevention practice PM. Physicians concerned with PM issues are generally interested in problems that have a significant impact on specific populations or those that affect one narrow segment of a population.[3]

PM physicians have to be skilled in the clinical practice of health promotion and prevention. They must understand the evidence–based medicine. Evidence–based medicine is a method to determine the content of clinical care, that involves the evaluation of the scientific evidence supporting a particular diagnostic test or therapy, and decides whether the evidence is sufficient to establish the efficacy and effectiveness of an intervention.[4]

Recently, the goal to concentrate on diseases and on the reduction of the risk factors has switched to the focus on actual behaviors that cause these conditions. By approaching threats to good health this way, behaviors such as smoking, unsafe sexual practices, dietary habits, and lack of exercise emerge as vitally important in determining disease or its absence. PM physicians embrace this approach to shape intervention strategy to target behaviors that cause disease.[5]

One tool of PM is the social marketing applied in health care

Social Marketing applies commercial marketing strategies to promote public health. Social marketing is effective on a population level, and health care providers can contribute to its effectiveness.[6]

Social Marketing is defined as ‘the application of proven concepts and techniques drawn from the commercial sector to promote changes in diverse socially important behaviors such as drug use, smoking, sexual behavior, etc’. This marketing approach has an immense potential to affect major social problems if we can only learn how to harness its power.[7]

Less attention is paid to the most important challenge any country might face regarding its population: changing behavior. In most instances, the problems posed by excessive drinking, unhealthy diets and the use of tobacco products and other harmful substances have more to do with a country's health status than do acute but short–lived illnesses.[8]

In 1985, the American Marketing Association created a niche for social marketing by expanding its definition of marketing by also including the promotion of ‘ideas’.[9]

In health care, social marketing still remains an elastic and elusive term. Often, social marketing is confused with mass media campaigns that seek to shape attitudes, increase awareness and encourage either the use of certain services or changes in personal or collective behavior. But, social marketers affirm that this specialty encompasses elements based on social advertising, that focus on messages applied differently.[8]

The first expansion of the social marketing  was the social communication, which focused on the message content to promotion through channels including personal selling, publicity and promotional events.[10]

The second expansion is called the social mobilization, a term used by UNICEF to stress out a political coalition and a community action.[11]

Social marketers can face several challenges in numbers and types of health issues competing for the public's attention; limitations on people's time; and increased numbers and types of communication channels, including the internet.[12]

The most important task is the segmentation of the audience, whether to apply a mass message or whether to ‘segment’ the message into target audiences. Audience segmentation is based on cultural, socio–demographic, behavioral characteristics that may be associated with the change behavior; called health lifestyle clusters.[13]

Targeted communication is an approach in which information about certain population groups are used to elaborate messages that draw attention to a specific segment.

‘Tailored’ communication is a more specific, individualized form of message, which can generate highly personalized messages. Such communications have been known as “any combination of information and behavior change strategies intended to reach one specific person, based on characteristics that are unique to that person, related to the outcome of interest, and derived from an individual assessment.[14] Because tailored communications are more precise and focus on specific cognitive and behavioral characteristics, they are more limited in the research of certain segments and more expensive to develop and to implement.

**Figure 1:**
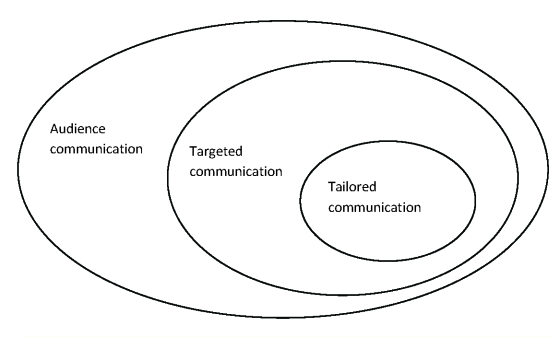
Types of communications

However understanding the targeted groups is a key element in developing a suited message. For each important market segment identified, formative research techniques are important to determine what motivates these people, what they desire for their health and what stands in their way. This means studying their lifestyle, cultural and media environment in order to determine what message will capture their attention, when, how and why.

Social marketing messages can aim to prevent risky health behaviors through education or the promotion of behavioral alternatives. Social marketing aimed at changing health behavior encounters external and internal competition. Digital communications proffer countless unhealthy eating messages along with seductive lifestyle images associated with cigarette brands. Cable television, the web, and video games offer endless opportunities for comorbid behavior. At the same time, products marketers add cigarettes or obscure benefits of foods (such as low salt content in foods high in saturated fat) to the confusion by marketing ‘reduced risk’.[8]

The evidence that social marketing is effective comes from research of mass communication campaigns. It's true that social marketing can change health behavior but in a very long time and the effects are often small.[15]

## Background

During 2008–2009, several PM programs have been developed and applied in Romania. These social programs had as main subjects smoking, diets, caffeine and alcohol consumption and lack of physical exercises.

The campaigns started because of the alarming rates of people who smoke, drink and have problems with their physical health. Accordingly, in 2008, 40% of the Romanians with ages ranging from 10 to 19 years old were smoking.[16] In 2009,  more than 60% of the adolescents over 18 years old were smoking.[17] Moreover, 33 000 Romanians die annually due to diseases aggravated by their smoking habit.[18] As a result, the Ministry of Health has taken the following measurements of prevention:

- the Ministry of Health has launched in 2007 the program ‘Stop Smoking’, through which it assured free counseling and treatment for tobacco consumers.
- on the 1^St^ of July 2008 the Ministry of Health has launched the national program ‘Smoking Kills’. Shocking images containing results people might get if they continue smoking were labeled on the wrappers of the tobacco products: 14 pictures show the risks of smoking envisaging lung and tongue diseases and premature death.
- in the first half of 2009, the Ministry of Health and the Ministry of Finance approved to raise the tobacco excises and introduce the vice tax in order to discourage the tobacco consumption. However, the tobacco consumption has not lowered.
- at the end of 2009, the Ministry of Health proposed a law through which people are not allowed to smoke in closed public places any more but only in specially equipped and ventilated rooms.

According to the ‘impact of pictograms on the packs of cigarettes on the adolescents in Romania’ study made by ‘Marius Nasta’ Institute of Pneumology, in 2009, on 1400 adolescents aged between 14 and 19 years old, the results were the following:

- 20% of the subjects smoke
- 6% have tried smoking
- 51% were disgusted by the images on the packs of cigarettes
- 45% are worried about the effects of smoking.

Regarding the caffeine consumption, in 2009, 63.5% of the adolescents drank coffee and Romania is currently situated on the 49^th^ place in the world in coffee consumption.[19]

As far as the alcohol consumption is concerned, 75% of the Romanian adolescents have occasionally consumed alcohol, according to a 2009 study. 28% of these admit to have consumed alcohol excessively.[20]

Moreover, the aliments consumption in Romania has raised in the last 3 years for the fast–food type. 2 out of 5 adolescents prefer eating more than 3 times a day in fast–foods.[21]

As a result of these alarming results The National Council of Audiovisual in Romania has launched, on the 29^th^ of September 2009, a campaign called ‘A healthy lifestyle’. This campaign is still running advertisements containing the following messages on TV and radio between 06:00 am and 10:00 p.m.:

- For a healthy life, consume daily fruits and vegetables;
- For a healthy life, exercise at least 30 minutes per day;
- For a healthy life, consume daily minimum 2 liters of liquids;
- For a healthy life, respect the main meals of the day;
- For a healthy life, avoid excessive consumption of salt, sugar and fats.

## Meth

The primary study was made on 304 students, average age 19, from ‘Carol Davila’ University of Medicine and Pharmacy in Bucharest, Romania, during one month, from 25 October to 25 November 2010. In order to make the primary study, a questionnaire was conceived and distributed to the panel of respondents. The questionnaire contained some demographic and personal details, such as age, origin, gender and marital status. To determine the respondents' attitude towards several issues that are inserted in the prevention medicine discipline, certain questions were inserted. With regard to the date of the last medical consultation, the respondents had to choose between ‘less than 3 months’, ‘between 3–6 months’, ‘6–12 months’, ‘more than 12 months’, whether the respondents were registered to a family doctor, suffered from a chronic disease, etc. The other questions aimed at the personal health of each respondent regarding physical exercises; if they did exercises moderately, like walking or climbing the stairs; occasionally like less than 4 times a week about 30 minutes; frequently like more than 4 times a week for about 30 minutes;  if they were on a diet and if they ate too salty or excessively fat food; if they ate moderately or rarely and the number of meals per day; their level of caffeine per day, specifying the number of cups of coffee, black tea or Coke they drank; their level or alcohol consumption noted as excessive, medium, less, or not at all; and tobacco consumption. Their answers helped us determine whether there was a connection between certain habits of adolescents and preventive medicine and, implicitly, if the social marketing applied in health care had any impact on the adolescents in Romania.

The results were obtained by using Microsoft Excel 2007 and SPSS version 13.0 Software.

For our correlations, we used the Pearson test. The Pearson test is a correlation coefficient, which indicates the level of association between two variables. The values around +/– indicate a positive/negative, strong correlation, the values around [+/–] 0.6 and [+/–] 0.8 indicate a strong correlation, [+/–] 0.5 indicate a medium correlation and around [+/–] 0.1 and [+/–] 0.4 indicate weak correlations.

## Results

We have surveyed a panel of 304 respondents and we received a response rate of 100%.  The panel was made up of adolescents with the average age of 19, 79 males and 225 females.

**Figure 2:**
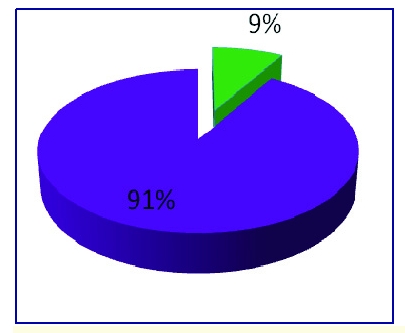
The distribution of sex rate

The average age rate was 19, taking into account the 224 responses with a 53.9% rate (164 respondents) followed by the ones aged 20 with a 8.6% rate (26 responses). 26.3% rate of the respondents did not specify their age (80).

**Figure 3:**
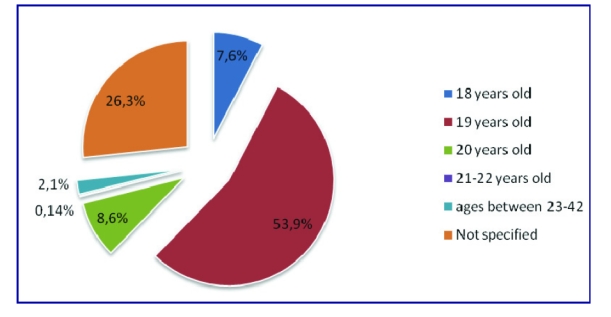
The distribution of age rate.

Out of the 304 subjects, 94 had a medical consultation in the last 3 months, 90 had a medical consultation between 3 and 6 months before, 61 had a medical consultation between 6 and 12 months before and 59 had their last consultation over 12 months before.

**Figure 4:**
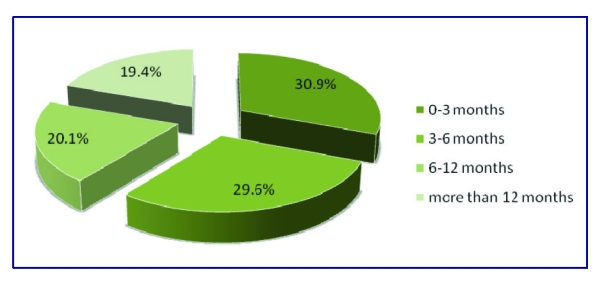
The distribution of last medical consultation rate.

284 adolescents are included in a family physician's evidence and only 20 responded that are not included in such a database.

**Figure 5:**
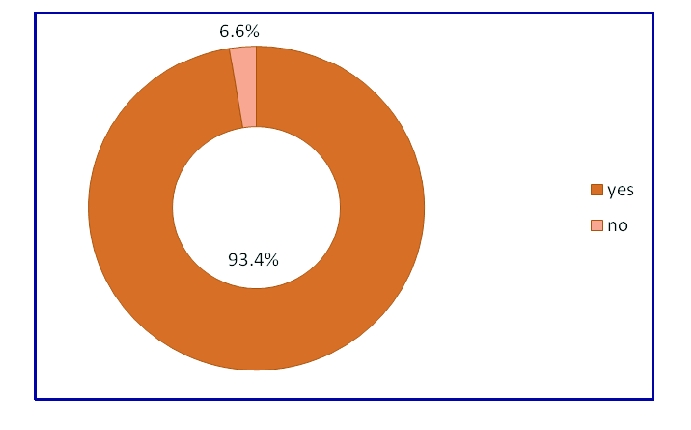
The percentage of the respondents who are included in a family physician evidence.

296 respondents have no chronic diseases, while 8 respondents have chronic diseases

**Figure 6:**
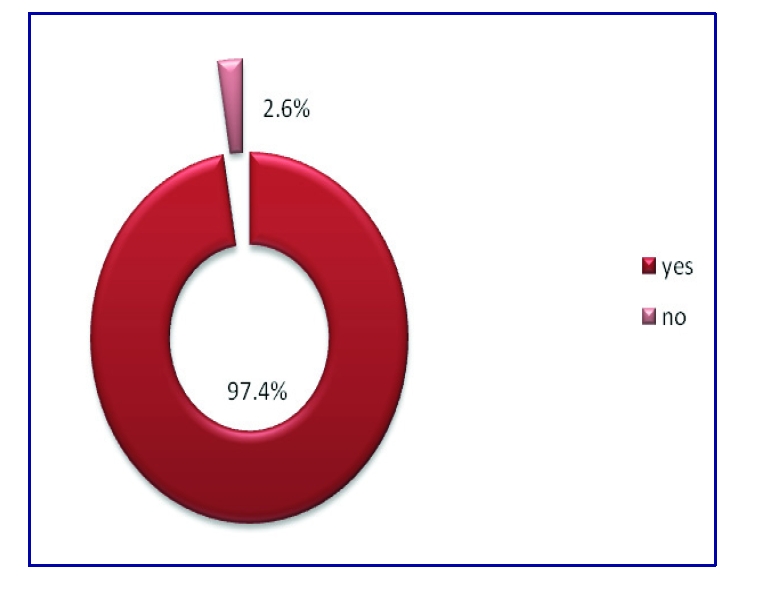
The chronic diseases percentages

7.2% of the respondents do workout exercises frequently, 34.9% of the respondents do workout exercises occasionally, 54.9% of the respondents do workout exercises moderately and 3% do not do workout exercises. 

**Figure 7:**
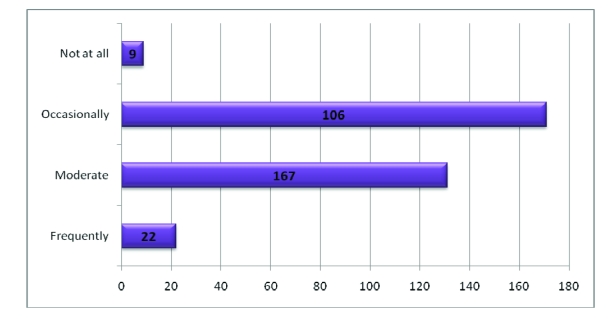
The number of subjects who do workout exercises

6.3% of the respondents declared that they are on a diet and 93.8% are not on a diet.

**Figure 8:**
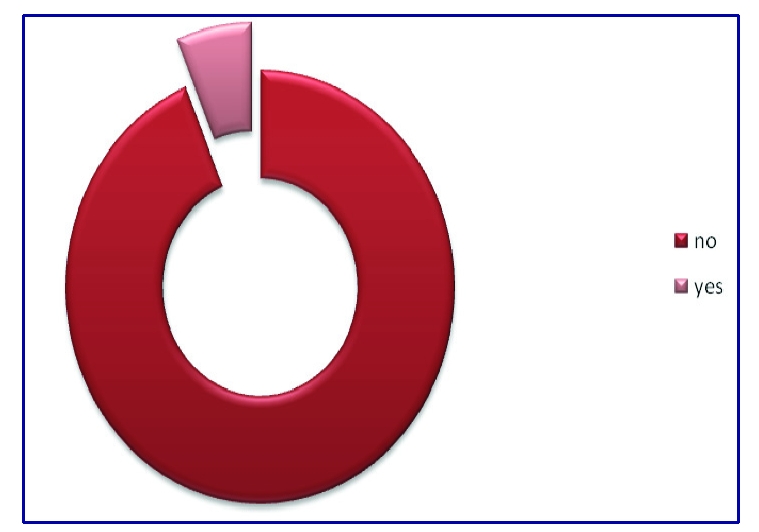
Percent of respondents who are on a diet

Regarding the number of meals they have per day, only 218 respondents answered. Therefore, 6 respondents have 1 meal per day, 64 respondents have 2 meals per day, 124 respondents affirmed that they have 3 meals per day, 14 respondents have 4 meals per day and 9 respondents have 5 meals per day. Accordingly, the average meals per day are 3.

**Figure 9:**
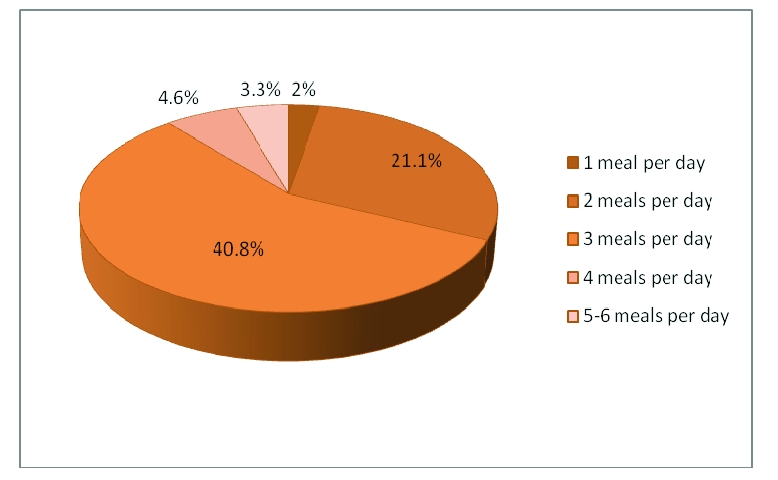
The rate of meals per day

Regarding the salty meals, 7.2% of the respondents answered that they eat excessively salty food, 55.9% of the respondents eat medium salty food and 36.5% of the respondents eat less salty food. Out of 304 respondents, only one did not indicate the level of salt of his meals. 

**Figure 10:**
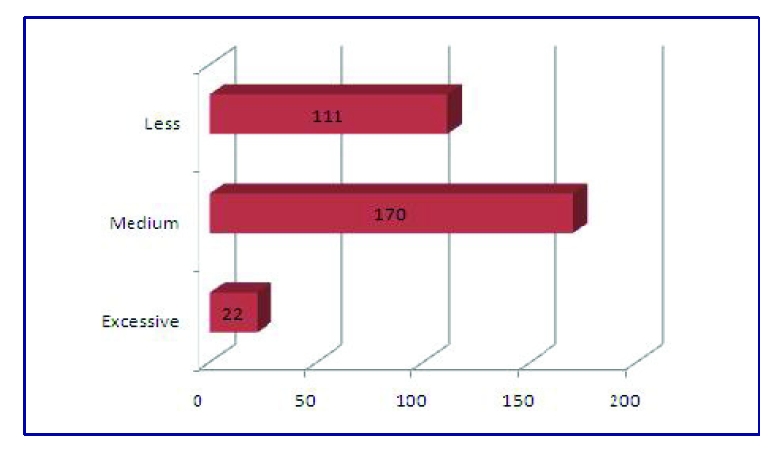
The number of adolescents who eat salty meals

Regarding fat meals, 0.7% of the respondents answered that they eat food excessively rich in fats, 43.1% of the respondents eat food moderately rich in fats, 56.3% of the respondents rarely eat food rich in fats.

**Figure 11:**
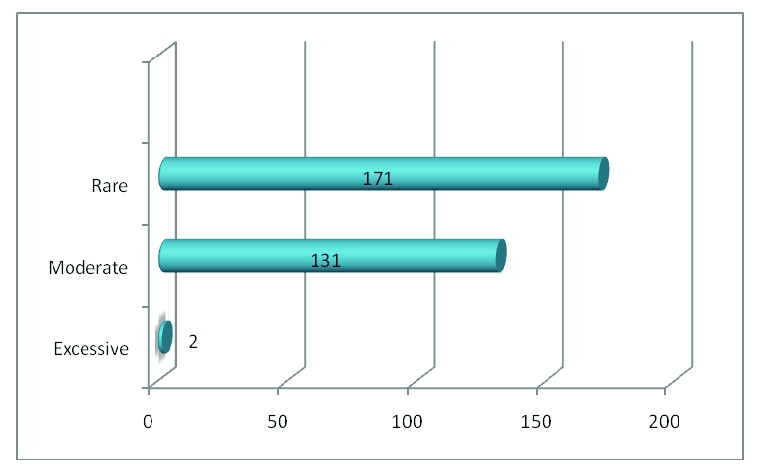
The number of respondents who meals rich in fats

According to the caffeine consumption, 54 respondents affirmed they do not drink caffeine beverages at all, 45 declared they only drink coffee, 76 declared they drink tea, 47 affirmed they drink Coke, 12 declared they drink only tea and Coke, 30 affirmed they consume coffee, Coke and tea, 15 declared they only drink coffee and Coke, 25 affirmed they drink coffee and tea.

**Figure 12:**
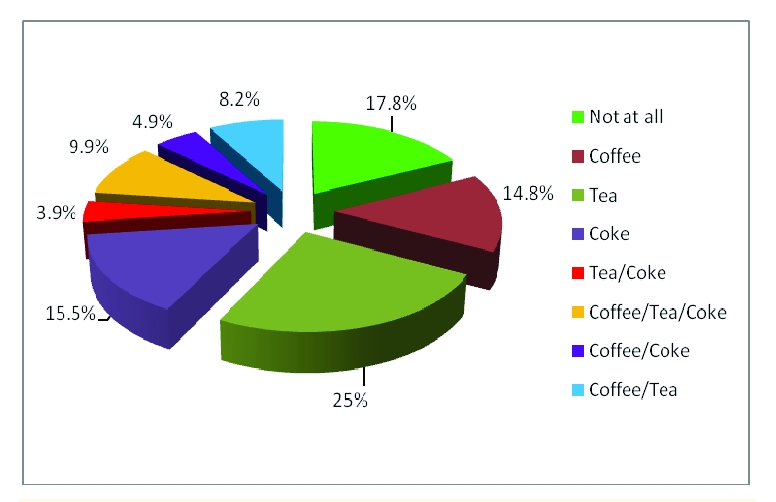
The rates for caffeine beverages

The rates for cups of caffeine per day, are as it follows: 17.8% do not drink any of the above –mentioned beverages, 26% respondents said they drink 1 cup per day, 21.1% said they drink 2 cups per day, 11.2% affirmed they drink 3 cups per day, 3.3% affirmed they drink 4 cups per day, 4.9% of all respondents affirmed they drink between 5 and 8 cups per day. Out of 304 respondents, only 84.5% responded and 15.5% did not answer. The average number of cups of caffeine beverages per day is two.

**Figure 13:**
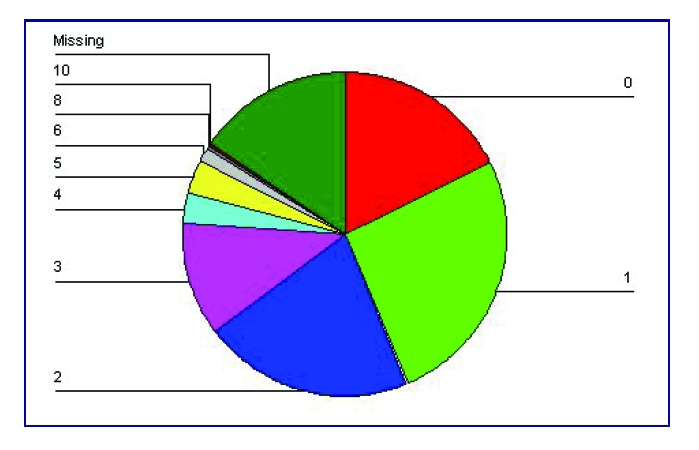
Number of caffeine cups per day

Out of 304 respondents (one respondent did not answer), 2 drink alcohol excessively, 17 drink alcohol medium, 133 drink little alcohol and 151 do not drink alcohol at all.

**Figure 14:**
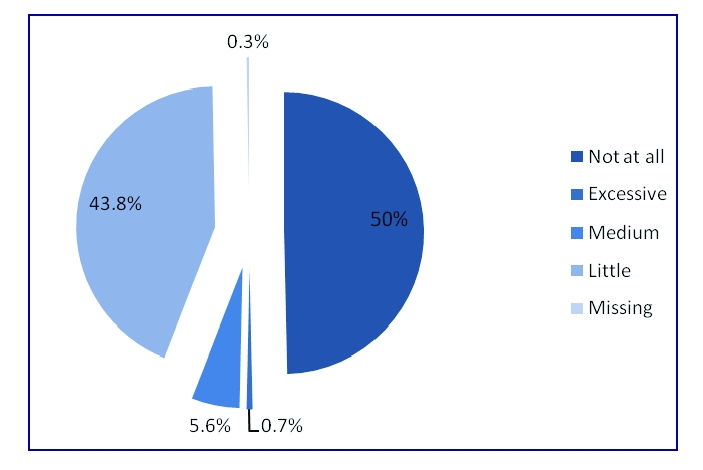
The alcohol percentages

58 respondents are smokers and 246 respondents are non-smokers.

**Figure 15:**
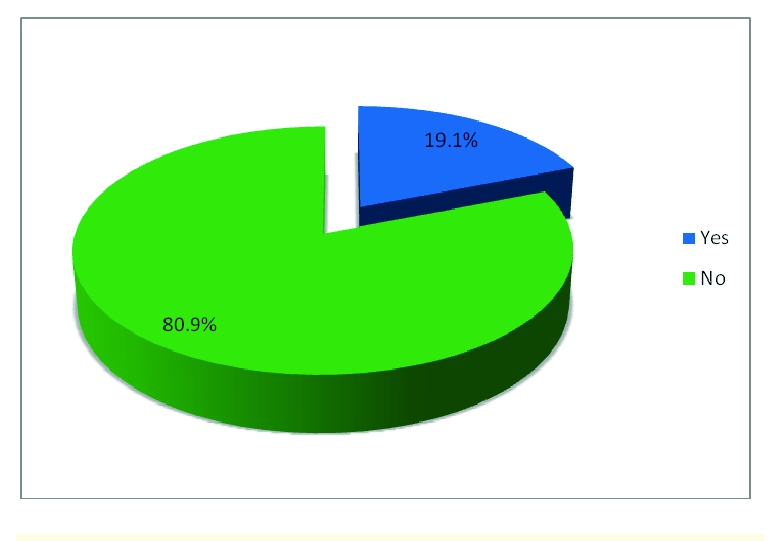
Percentages of tobacco consumption

The hypothesis that females tend to be on diets more than men do, was also tested. The t test for independent panels was used.  The results are shown in Table 1.

**Table 1:** Results of t test

Independent Samples Test									
	Levene's Test for Equality of Variances	T–test for Equality of Means							
	F	Sig	t	df	Sig. (2 tailed)	Mean difference	Std Error Difference	95% Confidence Interval	
								Lower	Upper
Equal variances assumed	11.017	0.001	1.588	302	0.113	5.02E–02	3.16E–02	–1.20E–02	0.11
Equal variances not assumed			2.004	229.801	0.046	5.02E–02	2.51E–02	8.56E–04	9.96E–02

After gathering all the necessary information and making all the relevant correlations, the main hypothesis of this paper was tested: The adolescents have a healthy life and the Prevention Medicine and the social marketing campaigns do not influence them.

A more exact hypothesis is the following: adolescents (students) who have an unhealthy life as far as the consumption (above average) of alcohol, tobacco, caffeine, salty and fat meals are concerned, do not apply the principles of Preventive Medicine.

The experimental model is the following:The raise of the unhealthy life (above average) leads to low preventive medicine (below average).

After applying the questionnaire on the panel, several data about its distribution were obtained. The mean, median and mode indicators have the convergent values of 3.35, 2 and 3. The standard deviation is 1.16 and the distribution elements are the skewness and the kurtosis, which have values lower than 1.

**Figure 16:**
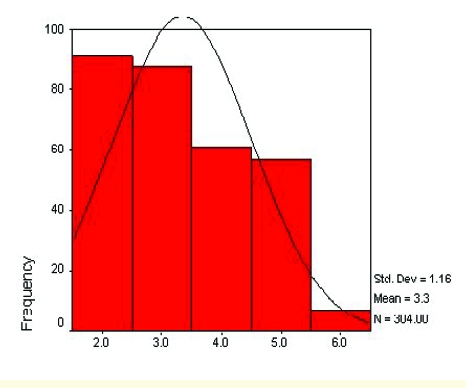
The distribution of the data panel

According to the above descriptive indicators, the distribution is homogenous, slightly asymmetric to the right.

The decision to test the main hypothesis with the Pearson correlation was taken because the following two main conditions are respected:

- the two variables are measures on an ordinal scale and
- the distribution of the two variables do not differ too much from the normal distribution.

When applying the Pearson correlation, a negative, not significant linear correlation, between the unhealthy life of adolescents and the low degree of the impact of preventive medicine on their behavior, was obtained. (r=0.04, p=0.42).

**Table 2:** The Pearson correlation results when testing the main hypothesis

Correlations			
		Preventive Medicine	Unhealthy Life
Preventive Medicine	Pearson Correlation	1.000	–0.046
	Sig. (2 tailed)		0.424
	N	304	304
Unhealthy Life	Pearson Correlation	–0.046	1.000
	Sig. (2 tailed)	0.424	
	N	304	304

## Discussion

This preliminary study included issues of diseases prevention among young respondents, and the influence of preventive medicine on their life style habits. The panel was made up of 304 respondents, adolescents with the average age of 19, mainly females, from Romania, belonging to the academic environment. The reason for choosing this panel was to see if preventive medicine had an impact on it, because they (the students) are more exposed to commercials, watch TV more than other possible respondents, and understand differently the spots and advertising campaigns warning them about preventing certain diseases. Due to the fact that 97.4% of the respondents are in the evidence of a family physician and had a medical consultation in the last 3 months, made us test the hypothesis that people who are in the database of a doctor have medical consultations frequently. According to the Pearson test of value 0.126 with p<0.05, there is a significant, positive, linear correlation between the two variables. 

Other correlations between several other variables were also tested. Accordingly, for the Pearson correlation of value –0.18(p<0.001), between workout exercises and sex, no linkage was found. Even if 93.8% of the respondents are not on a diet, they usually eat three non–salty non–fat meals a day, they do not drink too much caffeine or alcohol and they do not smoke. Despite this obvious facts, there was a very weak linkage between people who had a medical consultation 3 months before the questionnaire was applied and the fat meals consumed by the respondents (r=–0.119, p<0.038); between the ones who drink alcohol and smoke (r=0.247, p<0.001); and between the sex of the respondents and the tobacco consumption (r=<0.001, p=0.098).

Regarding the t test, made for the hypothesis that females tend to be on diets more than men do, the results were the following: because the condition of variance homogeneity of the two groups was not respected (the significance of Levene's test=0.01, being <0.05), we were compelled to choose the second line from Table 1, where t=2.004, df=229, p=0.046. This second option proved that the hypothesis is respected.

In conclusion, the fact that the significant level (p=0.01) was not reached while using the main hypothesis (Preventive Medicine and social marketing have no impact upon young students, with the average age of 19, belonging to the academic environment, in Romania), confirms that Preventive Medicine has no influence on the decisions taken by adolescents regarding their health, even if the results of the questionnaire showed encouraging facts.
